# Supplementary material for: Efficacy and safety of tibial cortex transverse transport for diabetic foot: A protocol for systematic review and meta-analysis
Source: PLoS One. 2022 Nov 4;17(11):e0277269. doi: 10.1371/journal.pone.0277269 (PMC9635701; doi:10.1371/journal.pone.0277269)
Supplement: Attachment — Submitted filename: Response to Reviewers.doc.docx [file pone.0277269.s002.docx]

Dear Reviewers:

Thank you for the valuable and helpful comments on our manuscript titled “**Efficacy and safety of tibial transverse transport for diabetic foot: a protocol for systematic review and meta-analysis**” (ID: PONE-D-21-40669). We have read your comments carefully and made corrections accordingly. We have uploaded the revised manuscript following the instructions provided in your letter. The revised sections are shown in red, and our responses to your comments are described below：

Reviewer #1:

1. **Comment: As the review of the specific topic, the paper seems to be superficial.** **Moreover, only the description of the protocol is not impressive. They did not show any results.**

Response: Thank you for pointing out this deficiency. We have revised and added content to the manuscript according to your advice. We defined diabetic foot (DF) and described pathological changes, epidemiological characteristics, and treatment status in the Introduction. Many traditional treatments are currently available for DF but have not achieved the expected results. As new technology, the tibial cortex transverse transport (TTT) technique has achieved satisfactory therapeutic results for DF. We defined TTT and discussed its advantages and relevant clinical studies that used this technology. In addition, the mechanism underlying TTT treatment for DF was systematically summarized. Many parts of the manuscript, such as eligibility criteria, information sources, search strategy, study selection, data extraction, assessment of study quality and publication bias, and statistical analysis, have been carefully revised and improved. We trust that these changes (shown in red) made the content more comprehensive and rigorous.

This paper is a protocol for systematic review and meta-analysis rather than original research. Data collection is ongoing, and relevant results will be published in the final meta-analysis. Literature on TTT treatment of diabetic foot is limited. This protocol can help fill knowledge gaps and establish guidelines for research design. Publishing research protocols allows researchers and funding agencies to keep abreast of research activities in related fields that are not yet widely known. In addition, these protocols improve research quality by reducing the number of duplicate studies and identifying deviations from the protocol during the study period. Please let us know if you have any questions or suggestions, and we will make corresponding modifications.

2. **Comment: The clear definition of TTT is required.**

Response: Thank you for your advice. TTT is a new technique for treating chronic lower-extremity angiopathy, such as that caused by diabetes. TTT has not been clearly defined to date. Notwithstanding, according to the most commonly accepted definition, TTT is based on Ilizarov’s "tension-stress rule" and natural rebuilding regeneration theory. Tibial draft produces tension stress, stimulating cell proliferation, stimulating the synchronous growth and repair of bones and associated muscle, fascia, blood vessels, and nerves, improving tissue microcirculation, and promoting the healing of leg and foot ulcers [11-12] (page 3-4, lines 64-68).

11. Ilizarov GA. The tension-stress effect on the genesis and growth of tissues: Part I. The influence of stability of fixation and soft tissuepreservation. Clin Orthop. 1989;238:249-281. <https://doi.org/>10.1097/00003086-198902000-00029 PMID: 2910611.

12. Chen Y, Kuang XC, Zhou J, Zhen PX, Zeng ZS, Lin ZX,et al. Proximal tibial cortex transverse distraction facilitating healing and limb salvage in severe and recalcitrant diabetic foot ulcers. Clin Orthop Relat Res. 2020;478(4):836-851. https://doi.org/10.1097/CORR.0000000000001075 PMID: 31794478

3. **Comment: The abbreviation is not adequately explained, for example, VEGF, bFGF, EGF, PDGF.**

Response: We are grateful for the suggestion. These abbreviations have been properly defined. The revised sections are shown in red. (page 5, lines 106-109).

4. **Comment:** **The description of** **reviewers, two reviewers, the third author seem to be odd as the subject in the manuscript writing.**

Response: We apologize for the misunderstanding. Division of labor for authors is usually described in the meta-analysis to avoid conflicts related to work allocation and author cooperation and improve workflow and work efficiency. We described the reviewers' work assignment according to the general habit of meta-analysis. Thank you for your careful review.

5. **Comment: English editing by a native is necessary.**

Response: Thank you for the suggestion. The manuscript was carefully revised by the authors for grammar and language issues and by Editage, a professional English language editing company. We trust that the methodology is better described in the revised manuscript. However, if you find other problems, please do not hesitate to let us know, and we will act accordingly to meet the standards of your journal.

Reviewer #2:

1. **Comment: The manuscript I received is incomplete. I found only abstract, introduction, statistical analysis, methods and discussion. I did not find the results or other data pertinent to the study. I suggest you submit the full manuscript for evaluation.**

Response: We apologize for the confusion. This paper is a protocol for systematic review and meta-analysis, rather than original research. Data collection is ongoing, and relevant results will be published in the final meta-analysis. This protocol can enable researchers and funding agencies to stay informed about research activities that are not widely publicized. These protocols improve research quality by reducing the number of duplicate studies. Moreover, full protocols provide more information than is required by trial registries, increasing research transparency and helping editors, reviewers, and readers identify deviations from the protocol during the study period.

Reviewer #3:

1. **Comment: In this review, the authors raise a new, comprehensive analysis to validate the beneficial treatment of tibial transverse transport (TTT) in patients with diabetic foot. This evaluation is valuable, since the current literature do not reach to a level with clear outcome indicators and limited search scope. I believe this review will bridge this cap. One issue the author may need to address is how TTT treatment effectively functions in the different stage of diabetic foot.**

Response: We appreciate your positive feedback. Our team is conducting clinical trials on TTT treatment of Wanger 3/4 diabetic foot to quantify the gene and protein expression of vascular endothelial growth factor (VEGF) and hypoxia-inducible factor 1-alpha (HIF-1α) during treatment and assess the effect of TTT on microvessel density (MVD) in diabetic foot wounds. Relevant manuscripts will be published after the completion of these experiments. We look forward to your suggestions.

We appreciate for reviewers’ warm work earnestly, and hope that the corrections will meet with approval. Some changes that we have made will not influence the content and framework of the paper, and we have thus not listed those changes, but marked them in red in the revised text. We trust that the revised manuscript is suitable for publication in your journal.

We look forward to hearing from you soon.

Sincerely,

Guanwen Sun

Email: [sunguanwen@sina.com](mailto:sunguanwen@sina.com)
