# Supplementary material for: Efficacy and safety of tibial cortex transverse transport for diabetic foot: A protocol for systematic review and meta-analysis
Source: PLoS One. 2022 Nov 4;17(11):e0277269. doi: 10.1371/journal.pone.0277269 (PMC9635701; doi:10.1371/journal.pone.0277269)
Supplement: Attachment — Submitted filename: Response to Reviewers.doc.docx [file pone.0277269.s003.docx]

Dear Reviewers:

Thank you for your invaluable comments regarding our manuscript titled “**Efficacy and safety of tibial transverse transport for diabetic foot: a protocol for systematic review and meta-analysis**” (ID: PONE-D-21-40669). Those comments were all valuable and very helpful for revising and improving our paper, as well as serving as important guidelines for our researches. We have studied the provided comments carefully and made corrections for which we hope will be met with approval. We have uploaded the revised manuscript following the instructions provided in your letter. The revised sections are shown in red, and our responses to your comments are included below:

**Response to Reviewers #1, #2, and #3:**

Thank you very much for your time and consideration regarding the publication of our paper. On behalf of my co-authors, I would like to express our great appreciation to the reviewers.

Reviewer #4:

1. **Comment: Define the outcomes that are not self explanatory. For example, I've never heard of "effective rate" before.**

**Response**: We are grateful for your suggestion. In our previous pre-search, we found that the outcome index "effective rate" appeared in several literatures [1-3]. In order to more completely evaluate the clinical effect of tibial transverse transport for diabetic feet, we included "effective rate" as the outcome index. Several other indicators are similar. We also agree with your suggestion, therefore, we will improve the phrasing of relevant outcome indexes in the subsequent formal study, and look forward to your further suggestions.

[1] Du SJ, Li YN, Lai JY. Clinical efficacy of modified tibial transverse transport in the treatment of diabetic foot. [Chinese Journal of Modern Drug Application](https://iffhce95c93b18bf148c3s5ubofkbvn0v96xw9fffi.res.gxlib.org.cn/perio/zgxdyyyy). 2021;15(5):29-31. DOI:10.14164/j.cnki.cn11-5581/r.2021.05.009

[2] Jin AL. Clinical analysis of tibial transverse bone transport in the treatment of

diabetic foot and lower extremity vascular occlusion. clinical research. [Do](https://iffhce95c93b18bf148c3s5ubofkbvn0v96xw9fffi.res.gxlib.org.cn/perio/dysh)ctor. 2020;5(15):26-28.https://iffhc975c3444c2dc4daas5ubofkbvn0v96xw9fffi.res.gxlib.org.cn/periodical/ChlQZXJpb2RpY2FsQ0hJTmV3UzIwMjIwODI0Eg1keXNoMjAyMDE1MDEwGghtam11dzFpZA%3D%3D

[3] Ma JW, Zhao LL, Ma DC. Analysis of clinical efficacy of treatment of diabetic foot of lateral tibial bone removal microcirculation regeneration technology. [Heilongjiang Science](https://iffhce95c93b18bf148c3s5ubofkbvn0v96xw9fffi.res.gxlib.org.cn/perio/hljkx). 2018;9(9):6-7. DOI:10.3969/j.issn.1674-8646.2018.09.003.
2. **Comment: Avoid fixed effect models since they under-perform in the presence of ANY heterogeneity. Random-effects (RE) models are more conservative and provide better estimates with wider confidence intervals:** **http://www.ncbi.nlm.nih.gov/pubmed/11252006 and http://www.ncbi.nlm.nih.gov/pubmed/21148194. What does the arbitrary 50% cut-off point add? Why is 49% fine and 51% an issue? My point is that a RE model will work better, in the presence of 5% heterogeneity, compared to a FE model!**

**Response**: Thank you for underlining this deficiency. This section was revised according to your advice. With numerous underlying variables in medical studies, establishing homogeneity is a rare commodity and some degree of variability between studies should be anticipated [1]. A random effects model, however, allows the true effect to vary across studies. Random effects model builds in an estimate of the between-study variation in effect size and typically provides wider overall confidence intervals [2]. Brockwell SE et al. thought that the random effects model should be adopted irrespective of the I^2^ heterogeneity statistic outcome [3]. Therefore, we are very happy to accept your suggestion and use the random effects model for statistical analysis (Line 217-221, page 11-12).

[1] Thompson SG, Pocock SJ. Can meta-analyses be trusted? Lancet 1991; 338(8775): 1127–1130.

[2] Kontopantelis E, Reeves D. Performance of statistical methods for meta-analysis when true study effects are non-normally distributed: A simulation study. Stat Methods Med Res. 2012;21(4):409-426.

[3] Brockwell SE, Gordon IR. A comparison of statistical methods for meta-analysis. Stat Med. 2001;20(6):825-840.

3. **Comment: Report the confidence intervals for I^2 (calculated using heterogi or metaan in Stata) as argued in** **http://www.ncbi.nlm.nih.gov/pubmed/17974687. A simple formula exists in the seminal 2002 Higgins paper that proposed** **I^2.**

**Response:** Thank you for your comment. We believe that all statistical tests for heterogeneity are weak, including I^2^. The clinical implications of this are considerable and must be examined on a case-by-case basis. Putting too much trust in homogeneity of effects may create a false sense of reassurance that one size fits all.  Given that I^2^ is not precise, 95% confidence intervals should always be given [1]. Therefore, we have set 95% confidence intervals for I^2^ (Line 222-223, page 12).

[1] Ioannidis JP, Patsopoulos NA, Evangelou E. Uncertainty in heterogeneity estimates in meta-analyses. BMJ. 2007;335(7626):914-6. doi: 10.1136/bmj.39343.408449.80. PMID: 17974687; PMCID: PMC2048840.

4. **Comment: The authors argue that if heterogeneity is high they will not meta-analyse. Indeed, very high heterogeneity estimates according to some researchers mean studies should not be meta-analysed. However, I disagree with that assessment for numerous methodological reasons. First heterogeneity is not study size independent: smaller meta-analyses are more often homogeneous and larger studies are heterogeneous (for both methodological and practical reasons). So according to this mantra we will be filtering out the most valuable analyses, the ones that involve many studies, over the much smaller ones. In addition, random effects models can model that heterogeneity and account for it. Finally, large heterogeneity is the norm and it's great if it has been picked up and can be incorporated in the model. It is much more problematic when the underlying heterogeneity is not picked up and studies are "safely" combined under a homogeneity assumption. In other words, small meta-analyses of “homogeneous” studies are much more problematic than large “heterogeneous” ones as evidenced in this http://www.ncbi.nlm.nih.gov/pubmed/23922860. In smaller meta-analyses existing heterogeneity is just not picked up well enough. The uncertainty of the estimate becomes obvious if the CIs for I^2 are reported, as argued in <http://www.ncbi.nlm.nih.gov/pubmed/17974687.>**

**Response**: Our deepest gratitude goes to you for your careful work and thoughtful suggestions that substantially helped improve our paper. Until we have received your advice, we believed that the very high heterogeneity means that studies should not be meta-analyzed. However, after reviewing the relevant data, we found that our previous view was indeed wrong, and some valuable research may be filtered out. With numerous underlying variables in medical studies, establishing homogeneity is a rare commodity and some degree of variability between studies should be anticipated [1]. When the number of studies required for meta-analysis is small, we often observe or assume that the variance estimate between studies is zero, and in fact this homogeneity assumption may be erroneous. Kontopantelis E et al. found levels of unobserved heterogeneity in the Cochrane data which appeared to be high, especially for small meta-analyses [2]. Whenever heterogeneity is detected, we do not ignore it, and we try to find its source (Line 230-233, page 12).

[1] Thompson SG, Pocock SJ. Can meta-analyses be trusted? Lancet. 1991; 338(8775): 1127–1130.

[2] Kontopantelis E, Springate DA, Reeves D. A Re-Analysis of the Cochrane Library Data: The Dangers of Unobserved Heterogeneity in MetaAnalyses. PLoS ONE. 2013;8(7): e69930. doi:10.1371/journal.pone.0069930

5. **Comment:  In search strategy, clarify that you will** **search "from inception" until the end date.**

**Response:** Thank you for your advice. We have added the full search date to the manuscript according to your advice (Line 30-31, page 2).

6. **Comment: Some minor language corrections are needed.**

**Response:** Thank you for your careful review. We are very sorry for the mistakes in this manuscript and inconvenience they caused in your reading. The manuscript has been thoroughly revised and rewritten by Editage, a professional English language editing company, so we hope it can now meet the journal’s standard.

7. **Comment: Year may be worth considering in bias assessment, especially if you don't have enough studies for a formal test: <http://www.ncbi.nlm.nih.gov/>pubmed/25988604. With newer studies we would be more confident.**

**Response:** Thank you for the suggestion. The issue of publication bias in meta-analyses is often completely ignored. We have carefully read the literature you recommended. Kicinski Michal et al. found that publication bias is smaller in meta-analyses of more recent studies, indicating their better reliability, and supporting the effectiveness of the measures for reducing publication bias in clinical trials. In general, publication bias is larger in meta-analyses of older studies, indicating their lower reliability [1]. Publication year has an important impact on publication bias, and we hope to include more new studies in subsequent studies in order to reduce it.

[1] Kicinski Michal, Springate David A, Kontopantelis Evangelos. Publication bias in meta-analyses from the Cochrane Database of Systematic Reviews [J]. Stat Med, 2015;34:2781-2793.
8. **Comment: Clarify** **the weighting for the model for analysing** **dichotomous outcomes.** **Inverse variance (****IV for random effects) or Mantel Haenszel (MH)? Note that MH is traditionally a fixed effect approach and the random effects version in RevMan is an IV-MH hybrid method. Anyway, clarification on the weighting is needed.**

**Response:** We deeply appreciate your suggestion. Accordingly, we have added a more detailed interpretation regarding the weighting of the model for dichotomous outcome analysis. We choose inverse variance to study the dichotomous outcomes. The study confidence interval and the total confidence interval are both 95%. We have added this information to the corresponding position in the manuscript (Line 210-212, page 11).

9. **Comment: How will the random-effect model be implemented, i.e. how will heterogeneity be estimated? There are numerous ways to do so. Did they use the** **standard DerSimonian and Laird method? If so, please state so. Also there are better performing methods, for example please see <https://www.ncbi.nlm.nih.gov/>pubmed/28815652 (or <http://www.ncbi.nlm.nih.gov/>pubmed/23922860) and the metaan command in Stata where these are implemented (https://www.stata-journal.com/article.html?article=st0201).**

**Response:** We agree with the comment and have expanded the sentence in the revised manuscript as follows:

“It will be implemented using the standard DerSimonian-Laird (D-L) method (Line 221-223, page 12).

10. **Comment: Do you expect to have to use continuity corrections (adding 0.5 in cells to ensues** **zero event studies are included)? Please reflect on the recommendations in this paper:** **http://bmjopen.bmj.com/content/6/8/e010983.full and discuss or make changes in your analyses as appropriate. Maybe a sensitivity analysis is needed.**

**Response:** Thank you for your suggestion. Friedrich recommended including zero event studies in all meta-analyses for the benefit of providing conservative point estimates and increasing the study integrity [1]. Cheng et al. found that including zero event studies provided more accurate overall pooled estimates than excluding them when there was no true treatment effect [2]. According to your comment, we have added the suggested content to the manuscript as follows:

“...if conditions permit, a continuity correction factor will be added to each of the four cells of the 2×2 table for zero event studies, i.e., to the event and non-event in the treatment arms, and event and non-event in the control arms. The constant continuity factor will be 0.5 (Line 233-237, page 12).”

[1] Friedrich JO, Adhikari NKJ, Beyene J. Inclusion of zero total event trials in meta-analyses maintains analytic consistency and incorporates all available data. BMC Med Res Methodol 2007;7:5.

[2] Cheng J, Pullenayegum E, Marshall JK, Iorio A, Thabane L. Impact of including or excluding both-armed zero-event studies on using standard meta-analysis methods for rare event outcome: a simulation study. BMJ Open. 2016 Aug 16;6(8):e010983. doi: 10.1136/bmjopen-2015-010983. PMID: 27531725.

11. **Comment: Cochran Q (i.e. chi-square) is notoriously underpowered to detect heterogeneity, especially for small meta-analyses http://www.ncbi.nlm.nih.gov/pubmed/9595615. I would not use.**

**Response**: We are very grateful to you for pointing out this problem. Paul and Donner found the power of the Cochran's Q test to be lower than those of other homogeneity tests for odds ratios in *k* 2x2 tables [1]. Hardy and Thompson found that the power of the Cochran’s Q test for heterogeneity can be low, especially when the total available information is low, i.e., in the case of sparse data [2]. After consulting the relevant materials, we found that Cochran's Q test usually has low power for detecting heterogeneity. Therefore, we will not use it for testing heterogeneity in future studies.

[1] Paul SR, Donner A. Small sample performance of tests of homogeneity of odds ratios in *k* 2x2 tables. Stat Med. 1992 Jan 30;11(2):159-65. doi: 10.1002/sim.4780110203. PMID: 1579755.

[2] Hardy RJ, Thompson SG. Detecting and describing heterogeneity in meta-analysis. Stat Med. 1998 Apr 30;17(8):841-56. doi: 10.1002/(sici)1097-0258(19980430)17:8<841::aid-sim781>3.0.co;2-d. PMID: 9595615.

Thank you for your careful review. We really appreciate your efforts in reviewing our manuscript during these unprecedented and challenging times. We wish good health to you, your family, and community. Your careful review has helped make our study clearer and more comprehensive. Some changes that we have made will not influence the content and framework of the paper, and we have thus not listed them here, but marked them in red in the revised text. We trust that the revised manuscript will be suitable for publication in your journal.

We look forward to hearing from you soon.

Sincerely,

Guanwen Sun

Email: [sunguanwen@sina.com](mailto:sunguanwen@sina.com)
